# Metaphyseal sleeves in arthroplasty of the knee: A suitable tool in management of major metaphyseal bone loss

**DOI:** 10.1007/s00132-020-04008-1

**Published:** 2020-10-21

**Authors:** Christian Lycke, Dirk Zajonz, Alexander Brand, Torsten Prietzel, Christoph-E. Heyde, Andreas Roth, Mohamed Ghanem

**Affiliations:** 1 Klinik für Orthopädie, Unfallchirurgie und Plastische Chirurgie, Universitätsklinikum Leipzig, Liebigstr. 20, 04103 Leipzig, Germany; 2 Klinik für Orthopädie, Unfall- und Wiederherstellungschirurgie, Zeisigwaldkliniken Bethanien Chemnitz, Chemnitz, Germany

**Keywords:** Retrospective study, Revision arthroplasty, Osteoarthritis, knee, Complications, Aseptic loosening, Retrospektive Studie, Revisionsarthroplastik, Gonarthrose, Komplikationen, Aseptische Lockerung

## Abstract

**Background:**

This study examined the clinical outcome following revision arthroplasty of the knee joint and severe arthrosis with metaphyseal bone defects and instability using metaphyseal sleeves. We analyzed the results based on established scores and recorded the complications occurring on revision arthroplasty.

**Material and methods:**

Patients with revision arthroplasty of the knee and metaphyseal bone defects grade III according to the Anderson Orthopedic Research Institute (AORI) classification were included (16 patients, 9 females and 7 males). In all cases, surgery was performed using an endoprosthesis COMPLETE™ revision knee system with metaphyseal sleeves.

**Results:**

All patients had a significant reduction in pain level after revision surgery. The median HSS score in the cohort with primary arthroplasty was 84 and in the cohort with revision arthroplasty 73 and the KSS was 83 and 55, respectively. According to the HSS an excellent result was achieved by 50% of the patients in the primary arthroplasty group and 25% in the revision group. Only three patients were considered to have an insufficient result. Postoperative pain was significantly reduced in both groups. The median ROM was 112° flexion in the primary arthroplasty group and 95° in the revision group. An extension deficit was observed in three patients and four patients showed prolonged wound healing postoperatively (25%), which was treated conservatively and did not lead to septic changes.

**Conclusion:**

The use of metaphyseal sleeves in patients with bone defects is a suitable instrument with no negative impact on the outcome both in primary and revision arthroplasty. Further studies with larger study groups and analysis of long-term results after use of such endoprosthetic components should be conducted.

## Abstract (de)

**Hintergrund:**

Diese Arbeit untersucht das klinische Ergebnis nach Verwendung sog. Sleeves (Hülsen) in der aseptischen Revisionsendoprothetik des Kniegelenks sowie bei schwerster Arthrose mit metaphysären Knochendefekten und Instabilität. Die Ergebnisse wurden auf der Grundlage etablierter Scores analysiert und die aufgetretenen Komplikationen erfasst.

**Material und Methoden:**

Es wurden Patienten mit Revisionsendoprothesen des Knies und metaphysären Knochendefekten mit einem Klassifizierungsgrad III nach dem Anderson Orthopedic Research Institute (AORI) eingeschlossen (16 Patienten; 9 weiblich und 7 männlich). In allen Fällen wurde die Operation mit dem Endoprothesen-Revisionssystem COMPLETE™ mit metaphysären Sleeves durchgeführt.

**Ergebnisse:**

Bei allen Patienten konnte eine signifikante Reduktion der Schmerzen durch die Revisionsoperation erzielt werden. Der Medianwert des HSS-Scores lag bei 84 in der Gruppe der primär Operierten und bei 73 in der Gruppe mit Voroperationen, der Wert des KSS-Scores betrug 83 bzw. 55. Entsprechend des HSS-Scores erhielten 50 % der Patientengruppe der Erstoperierten ein „ausgezeichnetes“ Ergebnis und 25 % in der Revisionsgruppe. Nur 3 Patienten wurden insgesamt als „unzureichend“ eingestuft. Die postoperativen Schmerzen waren im Vergleich zu den präoperativen in beiden Gruppen signifikant reduziert. Der mediane Bewegungsumfang (ROM) betrug 112° Beugung in der primär operierten Gruppe bzw. 95° in der Revisionsgruppe. Bei 3 Patienten wurde ein Extensionsdefizit beobachtet und 4 Patienten zeigten postoperativ eine prolongierte Wundheilung (25 %), die konservativ behandelt wurde und nicht zu septischen Revisionseingriffen führte.

**Schlussfolgerung:**

Metaphysären Sleeves stellen bei Patienten mit Knochendefekten ein geeignetes Instrument dar, das sowohl bei Primäreingriffen als auch bei Revisionsoperationen nicht zu unterlegenen Ergebnissen bei Patienten mit schweren metaphysären knöchernen Defekten führt. Weitere Studien mit größeren Studiengruppen und die Analyse der Langzeitergebnisse nach dem Einsatz solcher endoprothetischer Komponenten sollten durchgeführt werden.

## Introduction

With increasing numbers of primary total knee arthroplasty, the number of revision operations on the knee joint is increasing [6, 25], often due to infections, wear of modular parts, periprosthetic fractures or aseptic loosening [18, 20]. Periprosthetic osteolysis caused by polyethylene abrasion is one reason for bone defects, which are often seen in cases of loosening [10]. During revision surgery, explantation of components is usually accompanied by an increase of bone defects, especially in osteopenia or osteoporotic bones. In these situations, with significant bone loss in the metaphyseal part of the femur and/or the tibia, the exact positioning and permanent fixation of the revision components can be impaired. The management of these complicated cases can lead to longer operating times and thus to an increased risk of perioperative and postoperative complications [22]. Therefore, so-called metaphyseal sleeves are frequently used for the management of bone defects and have gained wide acceptance in revision arthroplasty of the knee [1]. These are cementless sleeves, which are anchored in the metaphysis of the femur and/or tibia as a modular part of the knee revision components. Combined with intramedullary stems, the sleeves are used to achieve a durable and stable anchorage situation in a deficient metaphyseal bone situation. The intramedullary stems are press-fitted into the medulla of the femur and/or tibia and provide additional stability in the diaphysis.

This study was carried out to examine the clinical outcome following revision arthroplasty of the knee joint and severe arthrosis with metaphyseal bone defects and instability using metaphyseal sleeves. We analyzed the results based on established scores and recorded the complications occurring with revision arthroplasty.

## Material and methods

**Fig. 1:**
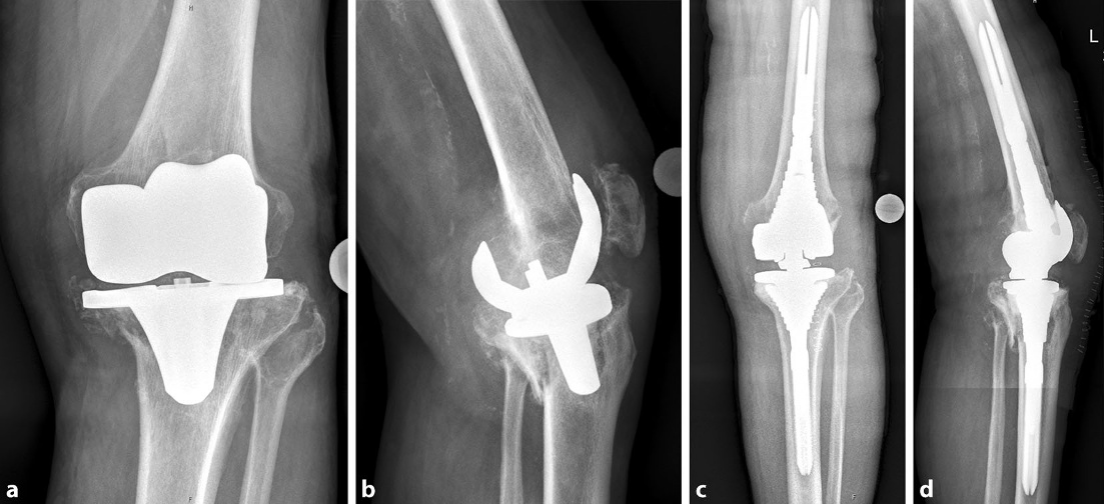
X‑ray radiographs (**a** anteroposterior and **d** lateral view) of a patient with aseptic KTEP loosening and simultaneous tibial and femoral periprosthetic fractures with metaphyseal bone loss after explantation of the primary components. Significant osteoporosis was identified during surgery (courtesy of the Department of Diagnostic and Interventional Radiology, University Hospital of Leipzig, all rights reserved). **c,** **d ** The same patient (**c** anteroposterior, **b** lateral view) after removal of the loosened total knee components and implantation of a rotating hinge knee revision system with metaphyseal sleeves (courtesy of the Department of Diagnostic and Interventional Radiology, University Hospital of Leipzig, all rights reserved)

**Fig. 2:**
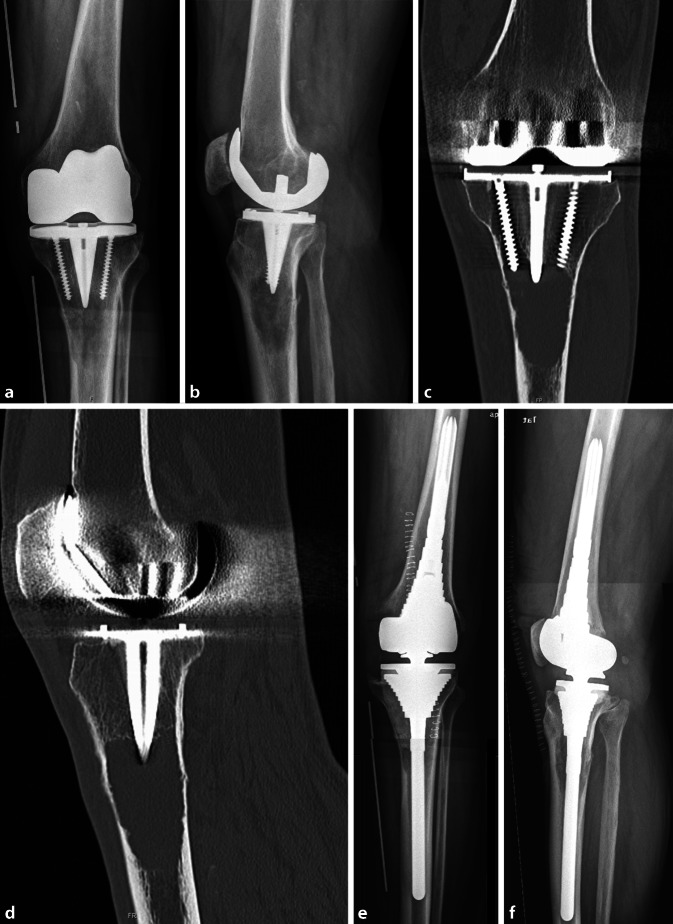
X‑ray radiographs (**a** anteroposterior, **b** lateral view) of aseptic loosening of primary total knee components with major osteolytic changes in the proximal tibial diaphysis due to wear of the inlay (particle disease) (courtesy of the Department of Diagnostic and Interventional Radiology, University Hospital of Leipzig, all rights reserved). **c,** **d ** Prior to surgery (**c** anteroposterior, **d** lateral view) a CT scan was performed to confirm the diagnosis and rule out malignant transformation (courtesy of the Department of Diagnostic and Interventional Radiology, University Hospital of Leipzig, all rights reserved). **e,** **f **The same patient (**e** anteroposterior, **f** lateral view) after implantation of a rotating hinge knee revision system with metaphyseal sleeves (courtesy of the Department of Diagnostic and Interventional Radiology, University Hospital of Leipzig, all rights reserved)

Prior to conducting this study a positive vote of the local ethics committee (Votum-No. 236/19-ek) was obtained. From May 2011 to March 2019, we identified patients who had undergone aseptic arthroplastic surgery of the knee with significant metaphyseal bone defects of the femur and/or tibia (Fig. 1) or major proximal diaphyseal defects due to inlay wear (Fig. 2). We evaluated patient data and conducted a clinical and radiological follow-up examination of the patients.

**Fig. 3:**
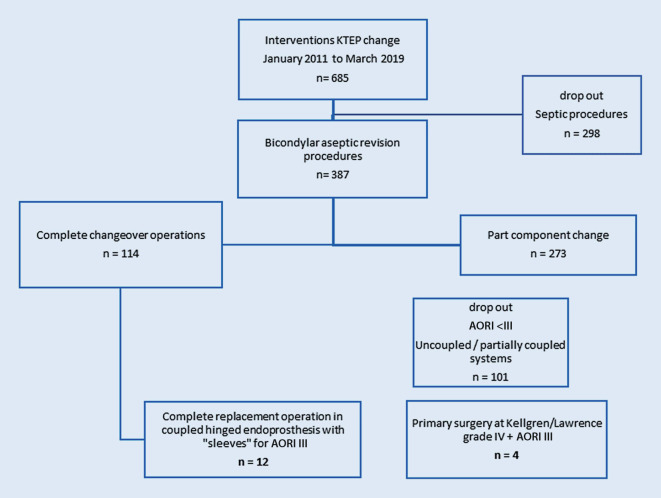
Overview of the patient selection process. Out of a total of 685 patients, 12 patients met the inclusion criteria, 4 patients with primary implants were additionally included *TKA* total knee arthroplasty, *AORI* Anderson Orthopedic Research Institute. All patients with primary surgery had the most severe grade IV arthrosis after Kellgren and Lawrence classification[17]

In order to have a homogeneous study group, we included patients with revision arthroplasty of the knee and metaphyseal bone defects grade III according to the AORI classification [9]. The major tibial and femoral bone deficiency situation and instability were intraoperatively confirmed as grade III. Patients with metaphyseal defects of class AORI I and II and patients with positive results for pathogens in the microbiological probe after explantation were excluded (Fig. 3). Furthermore, we included patients with primary varus gonarthrosis who had primary major metaphyseal bone defects due to severe osteoporosis along with significant deformity and hence were treated primarily with semi-constrained or constrained total knee arthroplasty.

Altogether 16 patients (9 females and 7 males) were included: 12 patients after revision arthroplasty of the knee and 4 patients with primary arthrosis along with severe metaphyseal bone defects and varus deformity. In all cases we performed surgery using an endoprosthesis COMPLETE™ revision knee system with the tibial and femoral metaphyseal sleeves LCS® valgus-varus-constrained VVC or S‑ROM® rotating hinge knee system (DePuy International, Leeds, UK). Clinical and radiological follow-up examinations were carried out. We evaluated the knee score according to Ranawat and Shine (HSS) and the functional and clinical knee society score according to Insall (KSS) [15]. Furthermore, we estimated the Numerical Rating Scale to compare the pain level and the preoperative and postoperative range of motion as well as the number of complications. The current radiological images were evaluated focusing on signs of loosening (e.g. radiolucent lines, changes of positioning). The data were evaluated with SPSS (IBM, Armonk, NY, USA) and Microsoft Excel 2019 (Microsoft, Redmond, WA, USA).

## Results

The average follow-up period was 79.5 months in the primary arthroplasty group and 31 months in the revision arthroplasty group. The average age at surgery of the patients was 76.5±12 years and 79 ±7 years, respectively (Tables 1 and 2). The median duration of surgery in the primary group was 259±26 min and in the revision group 151±57 min.

**Table 1:** Preoperative profiles of patients with a list of previous operations, age, pre-existing condition category (*ASA* classification according to the American Society of Anesthesiologists), duration of surgery and outcome (*ROM* range of motion, pain level and complications). The mean flexion was 95°. Almost every patient was able to perform a full extension (0°).

Patient	Age (years)	Previous knee surgery (*n*)	ASA	ROM (ex/zero/flex)	Pain level preoperative	Pain level postoperative	Complications	Operation duration (min)
1	92	1	3	0/0/90°	8	1	Recurring effusions	120
2	80	1	3	0/0/50°	7	3	Prolonged wound healing	275
3	68	1	3	0/30/120°	8	2	–	257
4	76	1	3	0/0/110°	8	2	–	80
5	90	1	3	0/0/30°	8	6	Prolonged pain	118
6	78	1	3	0/0/90°	10	6	Prolonged pain	158
7	74	1	2	0/0/100°	8	2	Recurring effusions	126
8	88	1	3	0/0/90°	8	5	Prolonged wound healing	147
9	75	7	3	0/5/50°	5	2	Retropatellar arthrosis	240
10	78	1	3	0/0/100°	5	0	Prolonged wound healing	151
11	87	1	3	0/0/100°	8	2	Postoperative hematoma	160
12	80	1	3	0/0/100°	7	1	Exacerbation of partial peroneal paralysis	151
Mean	79 ± 6.9	–	3	95°	8	2	–	151 ± 27.7

*Ex* extension, *flex* flexion, *zero* zero position

**Table 2:** Preoperative profiles of patients with primary operation, age, pre-existing condition category (*ASA* classification according to the American Society of Anesthesiologists), duration of surgery and outcome (*ROM* range of motion, pain level and complications)

Patient	Age (years)	ASA	ROM (ex/zero/flex)	Pain level preoperative	Pain level postoperative	Complication	Operation duration (min)
1	58	2	0/0/60°	8	4	Lymphedema	248
2	82	3	0/5/120°	6	0	–	270
3	71	3	0/0/115°	9	3	–	284
4	90	3	0/0/110°	10	2	Prolonged wound healing	215
Mean	76.5 ± 12	3	112.5°	8.5	2.5	–	259 ± 26

*Ex* extension, *flex* flexion, *zero* zero position

The median HSS score in the cohort with primary arthroplasty at the last time of examination (median follow-up 79.5 months, range 63–93 months) was 84 (±11) and in the cohort with revision arthroplasty 73 (±18) (median follow-up 31 months, range 24–94 months) and the KSS was 83 (±23) and 55 (±34), respectively. According to the HSS 50% of the patients achieved an excellent result in the primary arthroplasty group and 25% in the revision group. 25% received a “good” result respectively 33% in the revision group and 25% received a “mediocre” result respectively 17%. Only three patients were considered to have an insufficient result (Table 3).

**Table 3:** Overview of the outcome after surgery

Parameter	Median	Standard deviation	Range
*Flexion and pain levels in revision arthroplasty*			
HSS	73	±18.0	42–95
Clinical KSS	53	±24	5–87
Functional KSS	55	±34	0–100
ROM flexion	95°	±26.3°	–
Preoperative pain level	8	±1.3	5–10
Postoperative pain level	2	±1.9	0–6
Follow-up (months)	31	–	24–94
*Flexion and pain levels in primary arthroplasty*			
HSS	84	±11.0	66–95
Clinical KSS	72	±22	32–87
Functional KSS	82.5	±23	40–100
ROM flexion	112.5	±24°	–
Preoperative pain level	8.5	±1.4	6–10
Postoperative pain level	2.5	±1.9	0–4
Follow-up (months)	79.5	–	63–93

*HSS* Hospital for Special Surgery score system, *KSS* Knee Society score, *ROM* range of motion

**Fig. 4:**
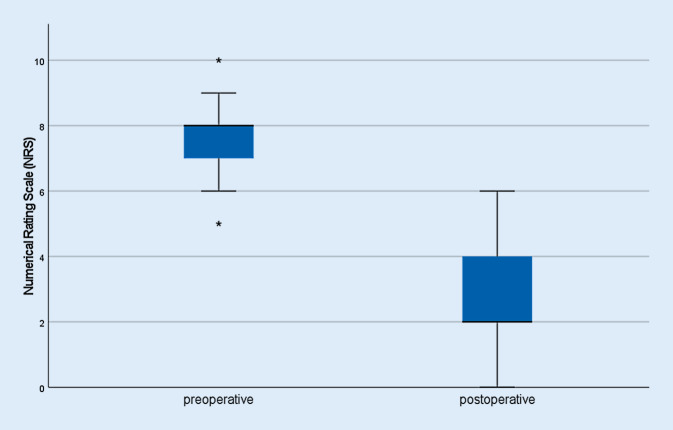
Overview of preoperative and postoperative pain levels using the numerical rating scale (NRS). Pain reduction was significant (*p* < 0.001): median preoperative pain level was 7.7/10 (±1.4) shown on the left side, median postoperative pain level was 2.7/10 (±1.9) on the NRS shown on the right side. The *Asterisk* represent the statistical outliers

Postoperative pain was significantly reduced in both groups compared to preoperative pain (2.7/10 ± 1.9 postoperative versus 7.7/10 ± 1.4 preoperative, *p* < 0.001) (Fig. 4). The median range of motion was 112° flexion (median ±24°) in the primary arthroplasty group and 95° (median; ±26.3°) in the revision group. An extension deficit was observed in three patients (18.75%, 30° − 5° extension deficit) and two patients (11.8%) showed postoperative swelling of the knee joint, of whom one suffered from chronic lymphedema (Tables 1 and 2).

Four patients had no specific postoperative complications (25%), two out of these four underwent previous knee surgery (50%). Four patients showed prolonged wound healing postoperatively (25%), which was treated conservatively and did not lead to septic changes. A postoperative hematoma had to be treated by puncture or surgical revision in two cases (12.5%). Of the patients two suffered from recurrent effusions until the last follow-up (12.5%). In one case (6.25%) a patellar tendon rupture occurred, which had to be treated with a patellar tendon graft 17 months after the initial revision operation. After 44 months the same patient received a retropatellar replacement due to retropatellar arthrosis. One patient (6.25%) with a known peroneal lesion showed increasing weakness of dorsiflexion and toe lifter from muscular strenght grade 3/5 (flexion against gravity feasible) according to Janda to 1/5 (muscular twitches) 14 days postoperatively and was treated conservatively. At the last follow-up the lesion was still present with only slight improvement (Janda 2/5 flexion under suspension of gravity feasible).

Two independent examiners found no radiological signs of aseptic loosening such as radiolucent zones, endoprosthetic shift or dislocation or localized cortical hypertrophy [16] in any of the cases (0%).

## Discussion

The most remarkable results of this retrospective follow-up examination were the significant postoperative pain relief and the absence of postoperative loosening. The average value of the HSS was 84 (±11) and in the cohort with revision arthroplasty 73 (±18) and can overall be considered as a good result. Rosso et al. obtained comparable results with an HSS of 82.5 (±8.4) [19]. The KSS was 83 (±23) and 55 (±34), respectively. Compared with reports in the literature, the results are similar to the survey by Graichen et al. (68.8 ± 23.3) [11] and Bugler et al. (58.1 ± 33.1) [7].

One specification of our study is the investigation of sleeve-stem systems in purely aseptic revision arthroplasty of the knee joint. According to our literature research, no prior study has exclusively evaluated the outcome after aseptic revision arthroplasty of the knee using metaphyseal sleeve-stem systems. The use of metaphyseal sleeves has already been investigated in other studies with different follow-up times, patient numbers and results with septic as well as aseptic cases ([2, 5, 8, 14]; Table 4). When comparing clinical and functional scores, the results of the present study are comparable with those of other studies [4, 12].

**Table 4:** Overview of comparable works and study results. The results of the HSS and KSS in this study were seperated into primary and revision TKA

Reference	Type of study	Patients (*n*)	Median follow-up (years)	Min. follow-up (years)	Max. follow-up (years)	HSS	Clinical KSS	Functional KSS
Alexander et al. [2]	Retrospective	28	2.8	2	4.3	–	94	–
Barnett et al. [4]	Retrospective	34	3.1	2	5.1	–	88.7 (±13.1)	75 (±18.7)
Bugler et al. [7]	Retrospective	35	3.2	2	5.1	–	81.3 (±18.1)	58.1 (±33.1)
Graichen et al. [11]	Prospective	121	3.6	2	6.2	–	147 (±32)	68.8 (±23.3)
Jones et al. [13]	Retrospective	15	4	2	6.1	–	134	–
Rosso et al. [19]	Retrospective	51	4.7	2	15.1	82.5 (±8.4)	79.6 (±24.9)	–
Guo et al. [12]	Retrospective	23	2.1	1	3.25	89 (±10.9)	–	–
This study	Retrospective	16	6.6 and 2.6	2	7.7	84 ± 11 and 73 ± 18	72 ± 22 and 53 ± 24	82,5 ± 23 and 55 ± 34

*HSS* Hospital for Special Surgery score system, *KSS* Knee Society score

The scores for HSS and KSS are comparable with the results of the listed studies; similar follow-up times are also shown

Another specific feature of our study is that all patients included in the study had severe metaphyseal bone defects of the AORI classification grade III [9, 21]. This distinguishes this patient population from other studies that included patients of all AORI grades in their study.

In the literature there are different results concerning septic or aseptic loosening of sleeve-stem systems. For example, results of the studies by Graichen et al., Bugler et al. and Watters et al. [7, 11, 24] showed loosening rates below 10%. Rosso et al. [19] documented a loosening rate of up to 41.5%. Graichen et al. [11] and Bugler et al. [7] had similar follow-up periods compared to this study (3.6 years, range 2–6.2 years and 3.2 years, range 2–5.1 years, respectively). Watters et al. [24] had a follow-up period of 5.3 years (range 2–9.6 years). Graichen et al. examined 121 patients, Watters et al. 108 and Bugler et al. 35; however, Rosso et al. [19] only used sleeves in patients with defects of AORI classification III, but also included patients with preoperative infections, which was an exclusion criterion in the present study. The absence of loosening in our work could then be an explanation of the fact that the pain level of the patients in this study was significantly lower after surgery was performed.

A limitation of this study lies in the small number of cases (*n* = 16); however, no study group with purely aseptic replacement surgery using sleeves for AORI III defects in revision knee arthroplasty has ever been investigated. Metaphyseal sleeves showed no negative impact on patient outcome: pain levels were significantly reduced (NRS preoperative 7.7 ± 1.4 vs. postoperative 2.7 ± 1.9; *p* > 0.001), the median range of motion was 112° and 95° flexion (median; ±27°), repectively and extension deficit was observed only in three patients (18.75%; 30° − 5° extension deficit). Two patients (12.5%) showed postoperative swelling of the knee joint and no patient suffered from postoperative instability. It is remarkable that patients with primary arthroplasty had a significantly longer operating time but, in the end, slightly better average scores in HSS and KSS and 50% of them showed no specific complication at all.

There are different ways of treating metaphyseal bone defects in revision arthroplasty of the knee, for example the use of bone cement for defect augmentation. This is only recommended for patients with AORI grades I and II [23]. Likewise, so-called wedges, bone chips or autologous bone blocks can be used as augmentation for bone defects, but these show only moderate long-term results [13]. Especially in the mentioned case 1 (Fig. 1) the use of a so-called metaphyseal cone would be another adequate option for treating metaphyseal defects like this [19]. These cones may be also useful to achieve a good metaphyseal fixation in the presence of poor bone quality. The choice for one or the other option depends on the surgeon’s experience, type, size and location of the defect and on the quality of the bone. For larger bone defects, the use of distal femoral replacement sets should be mentioned; however, the use of such megaimplants is associated with significantly higher intraoperative and perioperative complication rates [26].

Overall, the results of this study as well as comparable studies [3] indicate a positive benefit of metaphyseal fixation using sleeves in patients undergoing aseptic revision arthroplasty of the knee.

## Conclusion

Revision arthroplasty of the knee increasingly confronts patients as well as surgeons. Large metaphyseal bone defects are of particular significance. The use of metaphyseal sleeves in patients with bone defects is a suitable instrument, which has no negative impact on outcome both in primary arthroplasty and in revision arthroplasty. Further studies with larger study groups and analysis of long-term results after use of such endoprosthetic components should be conducted.

## Abbreviations

AORI: Anderson Orthopedic Research Institute
HSS: Hospital for Special Surgery score system
KSS: Knee Society Score
LCS: Low contact stress
NRS: Numerical Rating Scale
SPSS: Statistical Package for the Social Sciences
TKA: Total Knee Arthroplasty
